# Polymyxin B-immobilised fibre column treatment for acute exacerbation of idiopathic pulmonary fibrosis patients with mechanical ventilation: a nationwide observational study

**DOI:** 10.1186/s40560-023-00693-0

**Published:** 2023-10-11

**Authors:** Nobuyasu Awano, Taisuke Jo, Takehiro Izumo, Minoru Inomata, Yu Ito, Kojiro Morita, Hiroki Matsui, Kiyohide Fushimi, Hirokazu Urushiyama, Takahide Nagase, Hideo Yasunaga

**Affiliations:** 1 Department of Respiratory Medicine, Japanese Red Cross Medical Center, 4-1-22 Hiroo, Shibuya-Ku, Tokyo, 150-8935 Japan; 2 Department of Health Services Research, Graduate School of Medicine, The University of Tokyo, Tokyo, Japan; 3 Department of Respiratory Medicine, Graduate School of Medicine, The University of Tokyo, Tokyo, Japan; 4 Department of Clinical Epidemiology and Health Economics, School of Public Health, The University of Tokyo, Tokyo, Japan; 5 Department of Health Services Research, Faculty of Medicine, University of Tsukuba, Ibaraki, Japan; 6 Department of Health Policy and Informatics, Tokyo Medical and Dental University Graduate School of Medicine, Tokyo, Japan

**Keywords:** Idiopathic pulmonary fibrosis, Steroids, Polymyxin B-immobilised fibre column, Propensity score, Mortality

## Abstract

**Background:**

The prognosis for acute exacerbation of idiopathic pulmonary fibrosis (AE-IPF) is poor, and there is no established treatment. Hence, we aimed to investigate the effectiveness of a polymyxin B-immobilised fibre column (PMX) for the treatment of AE-IPF.

**Methods:**

Data were retrospectively collected from the Japanese Diagnosis Procedure Combination database from 1 July 2010 to 31 March 2018. We identified adult patients with idiopathic pulmonary fibrosis who received high-dose methylprednisolone (mPSL) therapy and mechanical ventilation upon admission. Eligible patients (n = 5616) were divided into those receiving PMX treatment combined with high-dose mPSL (PMX group, n = 199) and high-dose mPSL alone (mPSL alone group, n = 5417). To compare outcomes between the two groups, we applied a stabilised inverse probability of treatment weighting (IPTW) using propensity scores. The primary outcome was in-hospital mortality, and the secondary outcomes were 14- and 28-day mortality and length of hospital stay.

**Results:**

The in-hospital mortality rates of the PMX and mPSL alone groups were 79.9% and 76.4%, respectively. The results did not significantly differ between the two groups after performing a stabilised IPTW. The odds ratio of the PMX group compared with the mPSL alone group was 1.56 (95% confidence interval 0.80–3.06; *p* = 0.19). The 14- and 28-day mortality and length of hospital stay (secondary outcomes) also did not significantly differ between the two groups.

**Conclusions:**

In AE-IPF patients using mechanical ventilation, the treatment outcome was not significantly better for PMX combined with high-dose mPSL than for high-dose mPSL alone.

**Supplementary Information:**

The online version contains supplementary material available at 10.1186/s40560-023-00693-0.

## Background

Idiopathic pulmonary fibrosis (IPF) is an interstitial lung disease characterised by chronic and progressive fibrosis, with a poor prognosis and an average survival time of 3–4 years [1]. Patients with IPF sometimes develop acute respiratory failure, known as acute exacerbation of IPF (AE-IPF), which is associated with a 90-day mortality rate of approximately 50% [2].

In AE-IPF, various inflammatory mediators are produced and alveolar epithelial damage is induced, resulting in respiratory failure and pathological lesions of diffuse alveolar damage [1–4]. Although there is no established treatment for AE-IPF, the therapeutic options in the Japanese and international guidelines include immunosuppressive agents and corticosteroids, such as high-dose methylprednisolone (mPSL) [3, 4]. A polymyxin B-immobilised fibre column (PMX) is a medical device originally developed for removing circulating endotoxin and is used for treating sepsis [5, 6]. However, randomised trials did not show an effect of PMX for patients with sepsis [7]. Several studies have shown that PMX was associated with reducing several other inflammatory mediators, such as high-mobility group box 1 and neutrophils [8, 9]. Additionally, some reports have shown that PMX potentially could be effective for ARDS, which is similar in pathophysiology to AE-IPF [10, 11]. Small observational studies have shown that PMX treatment was associated with better short-term prognosis of AE-IPF [12–16]. However, these were retrospective studies with small sample sizes and lacked adjustment for confounding factors. Moreover, the effectiveness of PMX treatment in patients with AE-IPF who develop severe respiratory failure remains unclear.

This study used data collected from a Japanese nationwide inpatient database and aimed to evaluate the effectiveness of PMX treatment in patients with AE-IPF who developed severe respiratory failure.

## Methods

### Data source

Inpatient data were extracted from the Japanese Diagnosis Procedure Combination database, the details of which have been reported elsewhere [17]. More than 1000 hospitals, representing approximately 50% of all discharges from acute care hospitals in Japan. We collected data that included sex and age; hospitalisation and discharge dates; weight and height; severity of dyspnoea based on the Hugh–Jones dyspnoea scale [18]; level of consciousness upon admission; smoking index; activities of daily living; frequency of hospitalisation; intensive care unit (ICU) admission during hospitalisation; main diagnoses, pre-existing comorbidities upon admission and complications after admission as recoded by the attending physicians based on the International Classification of Diseases, 10th revision (ICD-10) codes accompanied by text in Japanese; procedures and their dates; dates and doses of drugs administered during hospitalisation; and discharge status.

### Patient selection

This study used data collected from 1 July 2010 to 31 March 2018. The inclusion criteria were patients aged ≥ 15 years, those diagnosed with interstitial pneumonia (ICD-10 codes J84.1, J84.8 and J84.9), those who underwent computed tomography scan within 1 day after admission and those who received treatment with intravenous mPSL at a dose of 500–1000 mg/day for 3 days, which was started within 4 days after admission [19, 20]. Patients with IPF were selected as follows. Firstly, we excluded patients with any of the following diagnoses of idiopathic interstitial pneumonias other than IPF recorded in Japanese text: idiopathic nonspecific interstitial pneumonia, respiratory bronchiolitis-associated interstitial lung disease, cryptogenic organising pneumonia, acute interstitial pneumonia, desquamative interstitial pneumonia, lymphoid interstitial pneumonia, idiopathic pleuroparenchymal fibroelastosis and unclassifiable idiopathic interstitial pneumonia. Secondly, we excluded patients with secondary interstitial lung diseases identified using ICD-10 codes (hypersensitivity pneumonitis [J67], connective tissue disease associated with interstitial lung disease [M05, M06 and M30–35], sarcoidosis [D86], amyloidosis [E85], drug-induced lung disease [J70], radiation pneumonitis [J70], *Pneumocystis jirovecii* pneumonia [B59], pneumoconiosis [J60–65], pulmonary alveolar proteinosis [J84.0)] eosinophilic pneumonia [J82], Langerhans cell histiocytosis [C96] and lymphangioleiomyomatosis [D21.9]); those receiving medications, including carperitide and tolvaptan for acute heart failure, within 1 day after admission; and those who received intra-aortic balloon pumping during hospitalisation [19, 20]. The remaining patients were assumed to have AE-IPF. Then, we also excluded patients with missing data about the level of consciousness, age and treatment year; patients who died within 6 days after admission to prevent immortal time bias; patients with sepsis (ICD-10 codes A40 and A41) and those without mechanical ventilation. In this study, we included only AE-IPF patients with mechanical ventilation under intubation and we did not count the use of non-invasive positive pressure ventilation as an inclusion criterion. Eligible patients were divided into two groups: those who received PMX treatment for ≥ 1 day, which was started within 6 days after admission, combined with high-dose mPSL (PMX group) and those who received high-dose mPSL alone (mPSL alone group).

### Characteristics of patients

The characteristics of patients evaluated in this study were sex, age, treatment year, body mass index, Hugh–Jones dyspnoea scale scores upon admission, level of consciousness upon admission, Charlson Comorbidity Index, smoking index, activities of daily living scale (Barthel Index) upon admission, history of previous hospitalisation (0, 1–2 or ≥ 3), type of hospital (academic or non-academic hospital), ICU admission and comorbidities. The Charlson Comorbidity Index scores were calculated according to the previous study (Additional file 1) [21]. The Charlson Comorbidity Index scores were classified into four categories (0, 1, 2 and ≥ 3). Further, we examined data on procedures and treatments, including haemodialysis, high-flow nasal cannula oxygen therapy and use of antibiotics and medications for IPF within 3 days after admission. We identified the use of hydrocortisone, as well as noradrenaline, as a treatment for shock, because shock is a complication indicating the severity in patients with AE-IPF. The Japan Coma Scale was used to evaluate the level of consciousness upon admission [22, 23], which is widely used in Japan and well correlated with the Glasgow Coma Scale score [24]. The ICD-10 codes were used to identify the following comorbidities (Additional file 2: Table S1) bronchial asthma, chronic obstructive pulmonary disease, pneumonia, pulmonary embolism, bronchiectasis, pneumothorax, lung and other types of cancer, disseminated intravascular coagulation, chronic heart failure, acute coronary syndrome, diabetes mellitus, stroke, renal failure, liver dysfunction, gastroesophageal reflux disease and urinary tract infection.

### Outcome

The primary outcome was all-cause in-hospital mortality. The secondary outcomes were 14- and 28-day mortality and length of hospital stay.

### Statistical analysis

Dichotomous and categorical variables were presented as numbers with percentages and continuous variables as the median and interquartile range (IQR). To account for differences in baseline characteristics between the two groups, we conducted stabilised inverse probability of treatment weighting (IPTW) analyses using propensity scores. Stabilised IPTW uses propensity scores and adjusts for measured potential confounders while preserving sample size [25]. To control covariate imbalance, the specific stabilised weights were generated using propensity scores, which can predict the probability of receiving PMX treatment combined with high-dose mPSL therapy. To estimate the propensity score, a logistic regression model for receiving high-dose mPSL alone therapy was used with the following independent variables: sex, age, treatment year, body mass index, Hugh–Jones dyspnoea scale score, level of consciousness upon admission, Charlson Comorbidity Index, smoking index, Barthel Index upon admission, frequency of hospitalisation, type of hospital, ICU hospitalisation within 3 days after admission, comorbidities and procedures (haemodialysis and high-flow nasal cannula oxygen therapy), antibiotics (ampicillin/sulbactam, tazobactam/piperacillin, third-generation cephalosporin, fourth-generation cephalosporin, carbapenem, fluoroquinolone and anti-methicillin-resistant *Staphylococcus aureus* drug) and drugs (noradrenaline, hydrocortisone, cyclophosphamide, tacrolimus, pirfenidone, nintedanib and furosemide). A standardised mean difference was used to assess covariate balance. A value of < 20% indicated an acceptable balancing of covariates between the two groups. Stabilised IPTW analyses can preserve sample size and appropriately estimate average treatment effects over the marginal distribution of measured covariates in a study cohort.

We used generalised linear models with cluster-robust standard errors, treating each hospital as a cluster, to compare the primary and secondary outcomes. Logistic regression analyses of in-hospital mortality and 14- and 28-day mortality were performed. Then, odds ratios and their 95% confidence intervals (CIs) were calculated. The lengths of hospital stay between the two groups were compared via Poisson regression analysis, and the incidence rate ratios and their 95% CIs were calculated. To address competing outcomes, the lengths of hospital stay was evaluated among the survivors alone and all patients.

We performed sensitivity analysis 1 for patients who received PMX treatment and/or high-dose mPSL therapy at an earlier stage after admission. We included patients diagnosed with interstitial pneumonia who received treatment with intravenous mPSL at a dose of 500–1000 mg/day for 3 days, which was started within 2 days after admission. Then, we divided the patients into two groups: those who received PMX treatment for ≥ 1 day, which was started within 4 days after admission, combined with high-dose mPSL (PMX_S1 group) and those who received high-dose mPSL alone (mPSL alone_S1 group). We excluded patients who died within 4 days after admission. Other inclusion and exclusion criteria were the same as in the main analysis. Furthermore, we conducted sensitivity analysis 2 which included only patients aged 51 years or older, because IPF develops predominantly in the elderly. We used the same inclusion and exclusion criteria as in the main analysis except for age, and divided patients into two groups: those who received PMX treatment combined with high-dose mPSL (PMX_S2 group) and those who received high-dose mPSL alone (mPSL alone_S2 group). We examined the same outcomes in sensitivity analysis 1 and 2 as in the main analysis.

A two-tailed significance level of 0.05 was used in all statistical analyses. STATA/MP version 16 software (STATA Corp., College Station, TX, USA) was used to perform all tests.

## Results

**Fig. 1:**
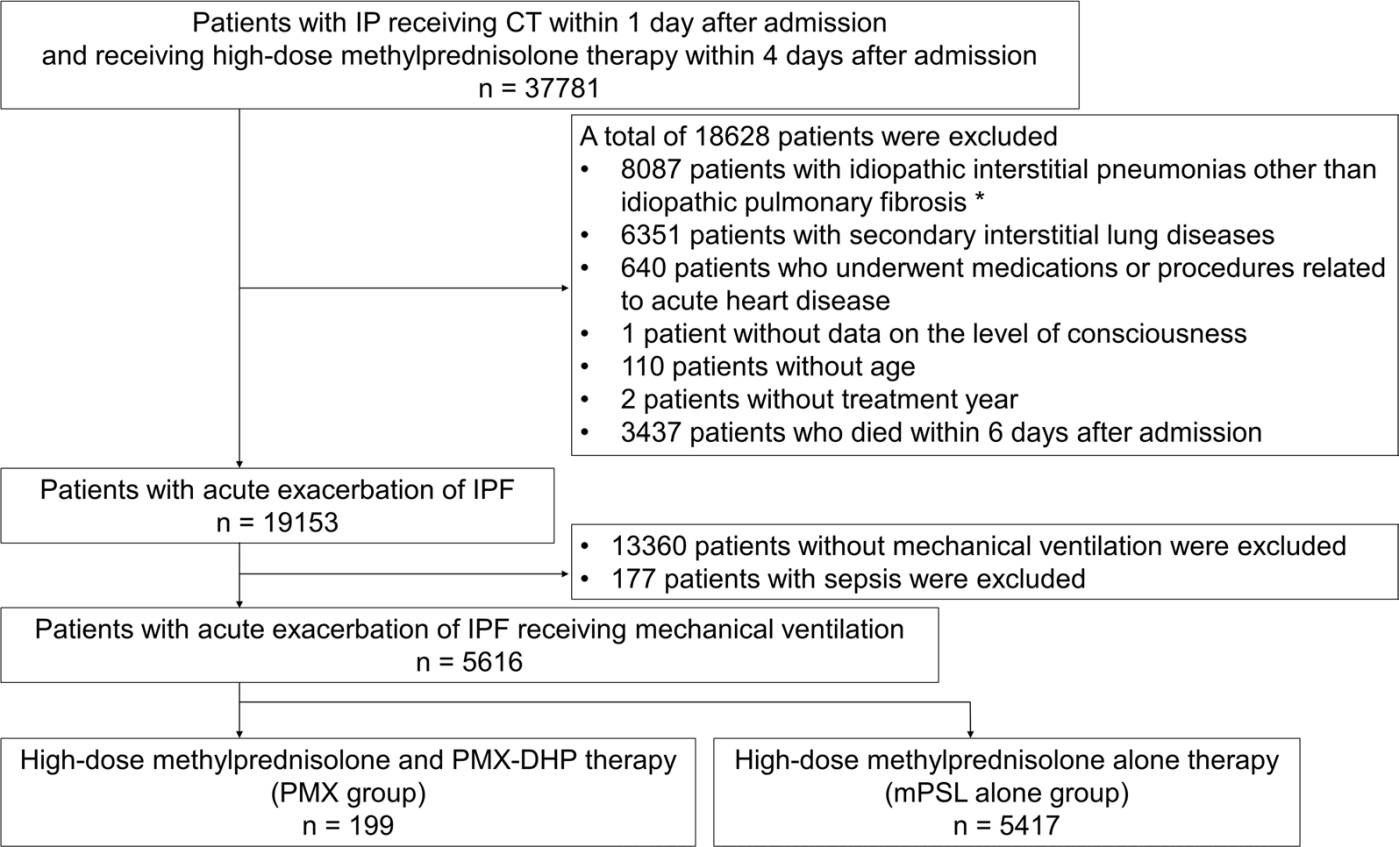
Flow chart of patient selection. *idiopathic nonspecific interstitial pneumonia, respiratory bronchiolitis-associated interstitial lung disease, cryptogenic organising pneumonia, acute interstitial pneumonia, desquamative interstitial pneumonia, lymphoid interstitial pneumonia, idiopathic pleuroparenchymal fibroelastosis and unclassifiable idiopathic interstitial pneumonia. IP, interstitial pneumonia; CT, computed tomography; IPF, idiopathic pulmonary fibrosis; mPSL, methylprednisolone; PMX, polymyxin B-immobilised fibre column

Figure 1 shows the process of patient selection. During the study period, 37781 patients underwent computed tomography scan within 1 day and received high-dose mPSL corticosteroid therapy within 4 days after admission. Among them, 5616 patients were eligible for this study. The patients were divided into the PMX group (n = 199) and the mPSL alone group (n = 5417). None of the patients in our cohort had undergone veno-venous extracorporeal membrane oxygenation or lung transplantation.

Table 1 shows the baseline patient characteristics, and Table 2 provides comorbidities and treatments before and after stabilised IPTW. The proportion of patients aged ≥ 80 years was higher in the mPSL alone group than in the PMX group. Moreover, the percentages of patients with BMI of < 23 kg/m^2^ and a higher Hugh–Jones dyspnoea scale scores were higher in the mPSL alone group than in the PMX group. A higher percentage of patients in the PMX (46.2%) group were admitted to the ICU. The percentage of patients with renal failure or receiving haemodialysis was higher in the PMX group than in the mPSL alone group. The frequency of some antibiotics use was not balanced between the two groups. After the stabilised IPTW using propensity scores, the baseline characteristics of the patients were well balanced between the two groups.

**Table 1:** Baseline characteristics of the patients before and after the stabilised IPTW using propensity scores

Characteristics	All patients					Patients after IPTW estimation				
	PMX group (n = 199)	%	mPSL alone group (n = 5417)	%	SMD	PMX group (n = 196)	%	mPSL alone group (n = 5435)	%	SMD
Male sex	153	76.9	4014	74.1	6.5	154	78.6	4035	74.2	10.3
Age, years										
15–70	79	39.7	1398	25.8	29.9	51	26.0	1430	26.3	− 0.8
71–80	85	42.7	2412	44.5	− 3.7	81	41.1	2420	44.5	− 6.9
≥ 80	35	17.6	1607	29.7	− 28.7	64	32.9	1585	29.2	8.1
Treatment year										
2010–2012	69	34.7	1604	29.6	10.9	61	31.2	1616	29.7	− 0.8
2013–2015	78	39.2	1924	35.5	7.6	68	34.5	1935	35.6	3.1
2016–2018	52	26.1	1889	34.9	− 19.1	67	34.3	1884	34.7	− 2.2
BMI (kg/m^2^)										
< 23	69	34.7	2695	49.8	− 30.9	94	48.2	2676	49.2	− 2.1
≥ 23	98	49.2	2082	38.4	21.9	84	43.1	2111	38.8	8.6
Missing data	32	16.1	640	11.8	12.3	17	8.7	648	11.9	− 10.5
Hugh–Jones dyspnoea score upon admission										
1–4	41	20.6	1319	24.3	− 9.0	63	32.2	1314	24.2	18.0
5	59	29.6	2532	46.7	− 35.7	79	40.5	2500	46.0	− 11.1
Missing data	99	49.7	1566	28.9	43.7	53	27.2	1620	29.8	− 5.7
Japan Coma Scale score upon admission										
0- or 1-digit (alert or dull)	189	95.0	5105	94.2	3.3	174	88.8	5122	94.2	− 19.4
2-digit (somnolence)	7	3.5	152	2.8	4.1	16	8.1	155	2.9	19.8
3-digit (coma)	3	1.5	160	3.0	− 9.8	6	3.1	158	2.9	0.9
Charlson Comorbidity Index										
0	116	58.3	2761	51.0	14.7	102	51.8	2791	51.3	0.9
1	33	16.6	658	12.1	12.7	18	9.4	666	12.3	− 9.2
2	36	18.1	1299	24.0	− 14.5	54	27.4	1289	23.7	8.5
≥ 3	14	7.0	699	12.9	− 19.7	22	11.4	689	12.7	− 4.1
Smoking index, pack-years										
0	76	38.2	2376	43.9	− 11.5	94	47.7	2374	43.7	8.1
1–39	40	20.1	1068	19.7	1.0	26	13.4	1070	19.7	− 16.9
≥ 40	50	25.1	1264	23.3	4.2	50	25.7	1271	23.4	5.4
Missing data	33	16.6	709	13.1	9.8	26	13.1	720	13.2	− 0.3
ADL upon admission (Barthel Index)										
100	47	23.6	1051	19.4	10.3	46	23.5	1065	19.6	9.6
≤ 95	113	56.8	3327	61.4	− 9.4	117	59.7	3329	61.3	− 3.1
Missing data	39	19.6	1039	19.2	1.1	33	16.7	1041	19.1	− 6.4
History of previous hospitalisation										
0	116	58.3	3140	58.0	0.7	109	55.9	3156	58.1	− 4.4
1–2	59	29.6	1645	30.4	− 1.6	74	37.6	1647	30.3	15.5
≥ 3	24	12.1	632	11.7	1.2	13	6.5	632	11.6	− 17.9
Academic hospital	163	81.9	4475	82.6	− 1.8	169	86.1	4493	82.7	9.1
ICU admission	92	46.2	1414	26.1	42.8	41	20.9	1469	27.0	− 13.0

Data are presented as n (%)

IPTW, inverse probability of treatment weighting; PMX, polymyxin B-immobilised fibre column; mPSL, methylprednisolone; SMD, standardised mean difference; BMI, body mass index; ADL, activities of daily living; ICU, intensive care unit

**Table 2:** Comorbidities and treatments before and after the stabilised IPTW using propensity scores

Variables	All patients					Patients after IPTW estimation				
	PMX group (n = 199)	%	mPSL alone group (n = 5417)	%	SMD	PMX group (n = 196)	%	mPSL alone group (n = 5435)	%	SMD
Comorbidity										
Bronchial asthma	10	5.0	259	4.8	1.1	6	3.1	260	4.8	− 7.8
Chronic obstructive pulmonary disease	6	3.0	292	5.4	− 11.8	5	2.4	287	5.3	− 14.5
Pneumonia	32	16.1	1033	19.1	− 7.9	40	20.2	1028	18.9	3.4
Pulmonary embolism	0	0.0	37	0.7	− 11.7	37	18.7	37	0.7	− 11.6
Bronchiectasis	4	2.0	149	2.8	− 4.9	5	2.6	147	2.7	− 0.9
Pneumothorax	2	1.0	41	0.8	2.7	1	0.5	42	0.8	− 2.4
Lung cancer	14	7.0	457	8.4	− 5.2	22	11.2	454	8.4	10.5
Other types of cancer^a^	7	3.5	408	7.5	− 17.6	11	5.7	400	7.4	− 7.4
Disseminated intravascular coagulation	39	19.6	473	8.7	31.5	30	15.3	506	9.3	17.3
Chronic heart failure	28	14.1	1125	20.8	− 17.7	38	19.3	1118	20.6	− 3.4
Acute coronary syndrome	9	4.5	404	7.5	− 12.4	15	7.6	398	7.3	1.3
Diabetes mellitus	48	24.1	1452	26.8	− 6.2	58	29.6	1450	26.7	6.7
Stroke	6	3.0	294	5.4	− 12.0	15	7.5	290	5.3	11.0
Renal failure	55	27.6	543	10.0	46.2	31	15.8	594	10.9	12.8
Liver dysfunction	9	4.5	276	5.1	− 2.7	13	6.4	276	5.1	6.3
Gastroesophageal reflux disease	15	7.5	687	12.7	− 17.1	24	12.4	677	12.4	− 0.2
Urinary tract infection	2	1.0	47	0.9	1.4	2	1.1	47	0.9	2.6
Treatment within 3 days after hospitalisation										
Haemodialysis	40	20.1	144	2.7	57.0	8	4.0	192	3.5	1.3
High-flow nasal cannula oxygen therapy	7	3.5	204	3.8	− 1.3	5	2.3	205	3.8	− 7.5
Ampicillin/sulbactam	18	9.0	745	13.8	− 14.8	31	15.9	737	13.6	7.5
Tazobactam/piperacillin	27	13.6	991	18.3	− 12.9	42	21.3	985	18.1	8.8
Broad spectrum β-lactam antibiotics^b^	133	66.8	2837	52.4	29.8	105	53.6	2880	53.0	1.3
Fluoroquinolone	99	49.7	1723	31.8	37.1	51	25.8	1772	32.6	− 14.0
Anti-MRSA drug	4	2.0	99	1.8	1.3	3	1.4	99	1.8	− 3.4
Noradrenaline	0	0.0	75	1.4	− 16.8	82	41.8	82	1.5	− 18.3
Hydrocortisone	3	1.5	119	2.2	− 5.1	2	0.9	118	2.2	− 9.1
Cyclophosphamide (intravenous)	17	8.5	138	2.5	26.4	5	2.8	155	2.9	− 0.5
Tacrolimus	3	1.5	51	0.9	5.1	1	0.6	53	1.0	− 3.5
Pirfenidone	7	3.5	93	1.7	11.3	1	0.6	96	1.8	− 7.4
Nintedanib	1	0.5	42	0.8	− 3.4	0	0.1	41	0.8	− 7.9
Furosemide	52	26.1	1720	31.8	− 12.4	67	34.1	1712	31.5	5.8

Data were presented as n (%)

IPTW, inverse probability of treatment weighting; PMX, polymyxin B-immobilised fibre column; mPSL, methylprednisolone; SMD, standardised mean difference; MRSA, methicillin-resistant *Staphylococcus aureus*

^a^Detailed information in Additional file 2: Table S1

^b^Third-generation cephalosporin, fourth-generation cephalosporin and carbapenem

The in-hospital mortality rates before the stabilised IPTW in the PMX and mPSL alone groups were 79.9% (159/199) and 76.4% (4137/5417), respectively (Tables 3). Tables 3 and 4 presents the outcomes after the stabilised IPTW. The in-hospital mortality rates of the PMX and mPSL alone groups were 83.7% (164/196) and 76.4% (4151/5435), respectively. The results did not significantly differ between the two groups, and the odds ratio of the PMX group was 1.56 (95% CI 0.80–3.06; *p* = 0.19). Similarly, the odds ratios of 14- and 28-day mortality in the PMX group were 1.16 (95% CI 0.58–2.31; *p* = 0.67) and 1.38 (95% CI 0.86–2.20; *p* = 0.18), respectively. In the PMX group, the incidence rate ratio of length of hospital stay was 0.94 (95% CI 0.78–1.13; *p* = 0.52) compared with that of the mPSL alone group, and the same results were obtained in the analysis of survivors alone.

**Table 3:** Outcomes in the PMX and mPSL alone groups before and after the stabilised IPTW

	Before the stabilised IPTW		After the stabilised IPTW	
	PMX group	mPSL alone group	PMX group	mPSL alone group
All patients, (n)	199	5417	196	5435
In-hospital mortality, n (%)	159 (79.9)	4137 (76.4)	164 (83.7)	4151 (76.4)
14-day mortality, n (%)	41 (20.6)	1152 (21.3)	47 (24.0)	1154 (21.2)
28-day mortality, n (%)	98 (49.2)	2645 (48.8)	111 (56.6)	2659 (48.9)
Length of hospital stay (days), median (IQR)	28 (16–55)	25 (15–45)	23 (15–38)	25 (15–45)
Survivor, (n)	40	1280	32	1284
Survival rate (%)	20.1	23.6	16.3	23.6
Length of hospital stay (days), median (IQR)	59 (34–87)	40 (25–62)	52 (31–79)	39 (25–62)

PMX, polymyxin B-immobilised fibre column; mPSL, methylprednisolone; IPTW, inverse probability of treatment weighting; IQR, interquartile range

**Table 4:** Comparison of outcomes between the PMX and mPSL alone groups after the stabilised IPTW

Logistic regression analyses of patients in the PMX and mPSL alone groups after the stabilised IPTW ^a^			
	Odds ratio	95% CI	*p* value
All patients			
In-hospital mortality	1.56	0.80–3.06	0.19
14-day mortality	1.16	0.58–2.31	0.67
28-day mortality	1.38	0.86–2.20	0.18

Incidence rate ratios of length of hospital stay in the PMX and mPSL alone groups after the stabilised IPTW^b^			
	Incidence rate ratio	95% CI	*p* value
All patients			
Length of hospital stay	0.94	0.78–1.13	0.52
Survivors			
Length of hospital stay	1.18	0.82–1.71	0.38

PMX, polymyxin B-immobilised fibre column; mPSL, methylprednisolone; IPTW, inverse probability of treatment weighting; CI, confidence interval

^a^The odds ratio of the PMX group compared to the mPSL alone group

^b^The incidence rate ratio of the PMX group compared to the mPSL alone group

The results of sensitivity analysis 1 restricted to patients who received PMX treatment and/or high-dose mPSL therapy at an earlier stage after admission were comparable to those of the main analyses (Additional file 3: Table S2, Additional file 4: Table S3, Additional file 5: Table S4, Additional file 6: Table S5). Similarly, the results of sensitivity analysis 2 for patients aged 51 years and older were consistent with those of the main analyses (Additional file 7: Table S6, Additional file 8: Table S7, Additional file 9: Table S8, Additional file 10: Table S9).

## Discussion

We used data from a nationwide database in Japan to investigate the effectiveness of PMX treatment combined with high-dose mPSL therapy in patients with AE-IPF. The results showed no significant difference in the in-hospital mortality between the PMX and mPSL alone groups. Similarly, the 14- and 28-day mortality and length of hospital stay did not remarkably differ between the two groups. The results of the sensitivity analyses supported these findings.

Several limited studies from Japan and Korea reported that PMX treatment improved the prognosis of AE-IPF [12–16]. Theoretically, PMX adsorbs inflammatory mediators, such as high mobility group box 1, neutrophils and interleukin-6 that cause respiratory failure in AE-IPF [8, 9, 26]. However, previous studies have been conducted only in a very small number of participating institutions and patients, and some studies lacked a control group [12–16]. Moreover, publication bias might also be a concern because there have been no studies of large populations since the first study showing the effectiveness of PMX treatment in AE-IPF was published in 2006 [11]. Using a national database, we examined the effectiveness of PMX treatment in a much larger number of IPF patients with respiratory failure than in previous studies and showed that no significant benefit was obtained from PMX with high-dose mPSL therapy compared with high-dose mPSL therapy alone.

When PMX treatment is administered to treat AE-IPF, it reportedly is more effective if started early after AE-IPF onset. Oishi et al. showed that prognosis was better for patients administered PMX treatment within 48 h of high-dose mPSL therapy than for those administered PMX treatment 48 h after high-dose mPSL therapy [27]. In our study, we included IPF patients who received treatment with high-dose mPSL, which was started within 2 days after admission for the sensitivity analysis 1. Patients in the PMX_S1 group were enrolled who received PMX treatment within 4 days after admission. However, the sensitivity analysis 1 also did not show significant effectiveness of PMX treatment for AE-IPF. However, our study was retrospective and included only AE-IPF patients with mechanical ventilation. Further studies are required to evaluate which populations of AE-IPF patients may benefit from PMX.

The mortality rate was higher in both groups in the current study than in previous ones [12–16] because our study included only AE-IPF patients with mechanical ventilation to reduce differences in disease severity between the two groups. Although the international guidelines for the diagnosis and management of IPF had a weak recommendation against the use of mechanical ventilation for IPF patients with respiratory failure [4], we often use mechanical ventilation for those patients in real-world clinical practice. Large-scale studies of AE-IPF patients with preserved respiratory condition who do not need mechanical ventilation are also needed in the future.

In the present study, we excluded patients with ICD-10 codes for sepsis. However, because our study included patients with AE-IPF who developed severe respiratory failure, we probably included patients with AE-IPF complicated by sepsis. To account for patients with sepsis, we compared outcomes of the two groups after balancing according to their treatments for severe infection. Furthermore, since our cohort included only patients with AE-IPF and the proportion of patients with sepsis was adjusted between the two groups, under the premise that PMX has no effect on sepsis, the effect of PMX may likely be on AE-IPF.

This study had several limitations. First, because the database did not include data about laboratory examinations, pulmonary function test results, performance status, the use of home oxygen therapy and radiological findings, the severity of IPF at AE onset could not be accurately evaluated. We only included patients who received mechanical ventilation to equalise the severity of AE-IPF between the two groups. In addition, baseline characteristics and treatments were well balanced between the two groups according to the stabilised IPTW. Second, although the IPF diagnosis was made by a physician, it was not confirmed by radiological and pathological examinations. Recently, multidisciplinary discussion (MDD) by physicians, radiologists and pathologists is recommended for the diagnosis of IPF [28]. In fact, however, it has been reported that not many facilities are able to perform MDD, and there is no established worldwide standardisation of the MDD or how to ensure its accuracy [29]. To accurately classify IPF, the diagnoses in the Japanese or ICD-10 codes were used to exclude all patients with idiopathic interstitial pneumonias other than IPF and secondary interstitial pneumonia because the specificity of respiratory disease diagnoses in the database is generally high [30, 31]. The strength of this study lies in the fact that we were able to accumulate a large number of cases, which would not have been feasible in a prospective study, and to evaluate the effectiveness of PMX using statistical methods. Third, although we included only AE-IPF patients who received mechanical ventilation, less than half of the original cohort were hospitalised in the ICU within 3 days after admission. The management of critical respiratory failure is multifaceted, and patient care outside the ICU may differ considerably from that inside the ICU. This divergence may reflect the unique situation in Japan. We conducted IPTW using propensity scores and balanced the frequency of ICU hospitalisation between the two groups.

## Conclusions

For the treatment of patients with AE-IPF who developed severe respiratory failure, PMX treatment combined with high-dose mPSL was not associated with better in-hospital mortality. Additional studies are required to evaluate the treatment of AE-IPF with PMX treatment.

### Supplementary Information

**Additional file 1.** The methodology for calculating the Charlson Comorbidity Index score.

**Additional file 2: Table S1.** List of ICD-10 codes used to identify comorbidities.

**Additional file 3: Table S2.** Baseline characteristics of the patients before and after the stabilised IPTW using propensity scores in the sensitivity analyses 1.

**Additional file 4: Table S3.** Comorbidities and treatments before and after the stabilised IPTW using propensity scores in the sensitivity analyses 1.

**Additional file 5: Table S4.** Outcomes in the PMX_S1 and mPSL alone_S1 groups before and after the stabilised IPTW in the sensitivity analyses 1.

**Additional file 6: Table S5.** Comparison of outcomes between the PMX_S1 and mPSL alone_S1 groups after the stabilised IPTW in the sensitivity analyses 1.

**Additional file 7: Table S6.** Baseline characteristics of the patients before and after the stabilised IPTW using propensity scores in the sensitivity analyses 2.

**Additional file 8: Table S7.** Comorbidities and treatments before and after the stabilised IPTW using propensity scores in the sensitivity analyses 2.

**Additional file 9: Table S8.** Outcomes in the PMX_S2 and mPSL alone_S2 groups before and after the stabilised IPTW in the sensitivity analyses 2.

**Additional file 10: Table S9.** Comparison of outcomes between the PMX_S2 and mPSL alone_S2 groups after the stabilised IPTW in the sensitivity analyses 2.

## Data Availability

The datasets used and/or analysed during the current study are available from the corresponding author on reasonable request.

## Abbreviations

AE: Acute exacerbation
CI: Confidence interval
DIC: Disseminated intravascular coagulation
ICD-10: International Classification of Diseases, 10th revision
ICU: Intensive care unit
IPF: Idiopathic pulmonary fibrosis
IPTW: Inverse probability of treatment weighting
IQR: Interquartile range
MDD: Multidisciplinary discussion
mPSL: Methylprednisolone
PMX: Polymyxin B-immobilised fibre column
