# Supplementary material for: Polymyxin B-immobilised fibre column treatment for acute exacerbation of idiopathic pulmonary fibrosis patients with mechanical ventilation: a nationwide observational study
Source: J Intensive Care. 2023 Oct 11;11:45. doi: 10.1186/s40560-023-00693-0 (PMC10568810; doi:10.1186/s40560-023-00693-0)
Supplement: Supplementary file 1 — Additional file 1. The methodology for calculating the Charlson Comorbidity Index score. [file 40560_2023_693_MOESM1_ESM.docx]

**Additional file 1**

**The methodology for calculating the Charlson Comorbidity Index score**

The Charlson Comorbidity Index is a method of predicting mortality by classifying or weighting comorbid conditions. To calculate this score, the following 12 diseases are identified as comorbidities in the patient. Each comorbidity is assigned a score, and the sum of the scores is the Charlson Comorbidity Index score.

Score 1: chronic pulmonary disease, rheumatologic disease, diabetes with chronic complications, renal disease

Score 2: congestive heart failure, dementia, mild liver disease, hemiplegia or paraplegia, any malignancy including leukaemia and lymphoma

Score 4: moderate or severe liver disease, acquired immunodeficiency syndrome or human immunodeficiency virus

Score 6: metastatic solid tumour
