# Supplementary material for: Polymyxin B-immobilised fibre column treatment for acute exacerbation of idiopathic pulmonary fibrosis patients with mechanical ventilation: a nationwide observational study
Source: J Intensive Care. 2023 Oct 11;11:45. doi: 10.1186/s40560-023-00693-0 (PMC10568810; doi:10.1186/s40560-023-00693-0)
Supplement: Supplementary file 3 — Additional file 3: Table S2. Baseline characteristics of the patients before and after the stabilised IPTW using propensity scores in the sensitivity analyses 1. [file 40560_2023_693_MOESM3_ESM.docx]

**Additional file 3**

**Table S2.** Baseline characteristics of the patients before and after the stabilised IPTW using propensity scores in the sensitivity analyses 1

|  | All patients | | |  | Patients after IPTW estimation | | |
| --- | --- | --- | --- | --- | --- | --- | --- |
| Characteristics | PMX_S1 group (n = 43) | mPSL alone_S1 group (n = 1509) | SMD |  | PMX_S1 group (n = 28) | mPSL alone_S1 group (n = 1539) | SMD |
| Male sex | 77% | 72% | 10.4 |  | 76% | 71% | 9.5 |
| Age, years |  |  |  |  |  |  |  |
| 15–70 | 30.2% | 24.3% | 13.3 |  | 25.1% | 24.2% | 2.2 |
| 71–80 | 65.1% | 42.7% | 46.2 |  | 59.6% | 44.2% | 31.1 |
| ≥80 | 4.7% | 33.0% | −77.8 |  | 15.3% | 31.6% | −39.2 |
| Treatment year |  |  |  |  |  |  |  |
| 2010–2012 | 30.2% | 29.2% | 2.4 |  | 33.8% | 28.6% | 11.1 |
| 2013–2015 | 39.5% | 36.8% | 5.7 |  | 43.9% | 37.7% | 12.6 |
| 2016–2018 | 30.2% | 34.1% | −8.2 |  | 22.4% | 33.7% | −25.4 |
| BMI (kg/m^2^) |  |  |  |  |  |  |  |
| <23 | 34.9% | 49.6% | −30.2 |  | 40.8% | 48.7% | −15.8 |
| ≥23 | 51.2% | 36.9% | 29.0 |  | 56.5% | 38.2% | 37.3 |
| Missing data | 14.0% | 13.5% | 1.5 |  | 2.7% | 13.2% | −39.4 |
| Hugh–Jones dyspnoea score upon admission | | | |  |  |  |  |
| 1–4 | 18.6% | 24.9% | −15.2 |  | 31.8% | 24.4% | 16.5 |
| 5 | 39.5% | 46.3% | −13.7 |  | 32.7% | 45.3% | −26.0 |
| Missing data | 41.9% | 28.8% | 27.5 |  | 35.5% | 30.4% | 11.0 |
| Japan Coma Scale score upon admission | | | |  |  |  |  |
| 0- or 1-digit (alert or dull) | 95.3% | 92.9% | 10.4 |  | 98.6% | 93.1% | 28.1 |
| 2-digit (somnolence) | 2.3% | 4.0% | −9.5 |  | 0.8% | 3.9% | −20.5 |
| 3-digit (coma) | 2.3% | 3.1% | −4.9 |  | 0.6% | 3.0% | −18.7 |
| Charlson Comorbidity Index | | | |  |  |  |  |
| 0 | 32.6% | 49.4% | −34.8 |  | 42.6% | 48.1% | −11.1 |
| 1 | 18.6% | 12.9% | 15.6 |  | 12.0% | 13.0% | −3.0 |
| 2 | 41.9% | 23.8% | 39.2 |  | 41.2% | 25.4% | 33.9 |
| ≥3 | 7.0% | 13.9% | −22.6 |  | 4.2% | 13.5% | −33.1 |
| Smoking index, pack-years | | | |  |  |  |  |
| 0 | 51.2% | 43.9% | 14.6 |  | 35.2% | 44.9% | −19.9 |
| 1–39 | 9.3% | 19.5% | −29.3 |  | 29.5% | 18.9% | 25.0 |
| ≥40 | 27.9% | 22.7% | 11.9 |  | 23.5% | 22.6% | 2.2 |
| Missing data | 11.6% | 13.9% | −6.9 |  | 11.8% | 13.6% | −5.5 |
| ADL upon admission (Barthel Index) | | | |  |  |  |  |
| 100 | 18.6% | 17.1% | 3.9 |  | 11.4% | 16.9% | −15.8 |
| ≤95 | 58.1% | 65.2% | −14.6 |  | 70.0% | 65.5% | 9.7 |
| Missing data | 23.3% | 17.7% | 13.8 |  | 18.6% | 17.6% | 2.6 |
| History of previous hospitalization | | | |  |  |  |  |
| 0 | 53.5% | 57.6% | −8.3 |  | 56.6% | 56.7% | −0.2 |
| 1–2 | 34.9% | 30.9% | 8.4 |  | 40.3% | 31.9% | 17.5 |
| ≥3 | 11.6% | 11.5% | 0.5 |  | 3.1% | 11.4% | −32.5 |
| Academic hospital | 88% | 82% | 18.0 |  | 80% | 82% | −5.9 |
| ICU admission | 51% | 26% | 54.2 |  | 47% | 27% | 41.4 |

Data are presented as n (%)

IPTW, inverse probability of treatment weighting; PMX, polymyxin B-immobilised fibre column; mPSL, methylprednisolone; SMD, standardised mean difference; BMI, body mass index; ADL, activities of daily living; ICU, intensive care unit
