# Supplementary material for: Polymyxin B-immobilised fibre column treatment for acute exacerbation of idiopathic pulmonary fibrosis patients with mechanical ventilation: a nationwide observational study
Source: J Intensive Care. 2023 Oct 11;11:45. doi: 10.1186/s40560-023-00693-0 (PMC10568810; doi:10.1186/s40560-023-00693-0)
Supplement: Supplementary file 4 — Additional file 4: Table S3. Comorbidities and treatments before and after the stabilised IPTW using propensity scores in the sensitivity analyses 1. [file 40560_2023_693_MOESM4_ESM.docx]

**Additional file 4**

**Table S3.** Comorbidities and treatments before and after the stabilised IPTW using propensity scores in the sensitivity analyses 1

|  | All patients | | |  | Patients after IPTW estimation | | |
| --- | --- | --- | --- | --- | --- | --- | --- |
| Variables | PMX_S1 group (n = 43) | mPSL alone_S1 group (n = 1509) | SMD |  | PMX_S1 group (n = 28) | mPSL alone_S1 group (n = 1539) | SMD |
| Comorbidity |  |  |  |  |  |  |  |
| Bronchial asthma | 9% | 6% | 13.1 |  | 4% | 6% | −6.2 |
| Chronic obstructive pulmonary disease | 7% | 5% | 7.2 |  | 5% | 5% | −1.3 |
| Pneumonia | 9% | 20% | −30.8 |  | 11% | 21% | −28.9 |
| Pulmonary embolism | 0% | 0% | −8.2 |  | 0% | 0% | −7.8 |
| Bronchiectasis | 0% | 3% | −23.6 |  | 0% | 3% | −23.9 |
| Pneumothorax | 2% | 1% | 12.9 |  | 1% | 1% | −0.9 |
| Lung cancer | 7% | 8% | −4.2 |  | 2% | 8% | −24.1 |
| Other types of cancer ^a^ | 7% | 7% | −1.7 |  | 5% | 7% | −8.2 |
| Disseminated intravascular coagulation | 12% | 7% | 15.8 |  | 17% | 7% | 34.7 |
| Chronic heart failure | 30% | 22% | 19.0 |  | 29% | 23% | 13.9 |
| Acute coronary syndrome | 5% | 7% | −11.6 |  | 1% | 7% | −26.2 |
| Diabetes mellitus | 9% | 23% | −36.9 |  | 31% | 22% | 26.0 |
| Stroke | 5% | 6% | −6.7 |  | 7% | 6% | 3.1 |
| Renal failure | 33% | 11% | 54.9 |  | 13% | 13% | 0.2 |
| Liver dysfunction | 9% | 5% | 16.5 |  | 11% | 5% | 21.1 |
| Gastroesophageal reflux disease | 7% | 10% | −9.9 |  | 7% | 10% | −9.5 |
| Urinary tract infection | 0% | 1% | −13.2 |  | 0% | 1% | −13.1 |
| Treatment within 3 days after hospitalisation | | | |  |  |  |  |
| Haemodialysis | 21% | 3% | 57.6 |  | 3% | 5% | −6.8 |
| High-flow nasal cannula oxygen therapy | 0% | 5% | −30.7 |  | 0% | 5% | −31.8 |
| Ampicillin/sulbactam | 19% | 14% | 13.8 |  | 14% | 15% | −2.1 |
| Tazobactam/piperacillin | 23% | 20% | 7.5 |  | 24% | 20% | 10.6 |
| Broad spectrum β-lactam antibiotics ^b^ | 63% | 52% | 21.0 |  | 37% | 53% | −32.9 |
| Fluoroquinolone | 35% | 32% | 5.2 |  | 34% | 32% | 4.7 |
| Anti-MRSA drug | 0% | 2% | −20.8 |  | 0% | 2% | −21.3 |
| Noradrenaline | 2% | 2% | 3.3 |  | 0% | 2% | −10.8 |
| Hydrocortisone | 0% | 2% | −21.5 |  | 0% | 2% | −20.9 |
| Cyclophosphamide (intravenous) | 2% | 3% | −4.8 |  | 0% | 3% | −16.8 |
| Tacrolimus | 0% | 1% | −11.0 |  | 0% | 2% | −36.2 |
| Pirfenidone | 2% | 2% | 5.3 |  | 0% | 2% | −9.9 |
| Nintedanib | 0% | 1% | −12.1 |  | 0% | 1% | −12.4 |
| Furosemide | 35% | 33% | 4.1 |  | 26% | 34% | −16.9 |

Data were presented as n (%)

IPTW, inverse probability of treatment weighting; PMX, polymyxin B-immobilised fibre column; mPSL, methylprednisolone; SMD, standardised mean difference; MRSA, methicillin-resistant *Staphylococcus aureus*

^a^ Detailed information in Additional file 2: Table S1

^b^ Third-generation cephalosporin, fourth-generation cephalosporin and carbapenem
