# Supplementary material for: Polymyxin B-immobilised fibre column treatment for acute exacerbation of idiopathic pulmonary fibrosis patients with mechanical ventilation: a nationwide observational study
Source: J Intensive Care. 2023 Oct 11;11:45. doi: 10.1186/s40560-023-00693-0 (PMC10568810; doi:10.1186/s40560-023-00693-0)
Supplement: Supplementary file 5 — Additional file 5: Table S4. Outcomes in the PMX_S1 and mPSL alone_S1 groups before and after the stabilised IPTW in the sensitivity analyses 1. [file 40560_2023_693_MOESM5_ESM.docx]

**Additional file 5**

**Table S4.** Outcomes in the PMX_S1 and mPSL alone_S1 groups before and after the stabilised IPTW in the sensitivity analyses 1

|  |  | Before the stabilised IPTW | |  | After the stabilised IPTW | |
| --- | --- | --- | --- | --- | --- | --- |
|  |  | PMX_S1 group | mPSL alone_S1 group |  | PMX_S1 group | mPSL alone_S1 group |
| All patients, (n) | | 43 | 1509 |  | 28 | 1539 |
|  | In-hospital mortality, n (%) | 38 (88.4) | 1240 (82.2) |  | 23 (82.1) | 1267 (82.3) |
|  | 14-day mortality, n (%) | 22 (51.2) | 736 (48.8) |  | 14 (50.0) | 762 (49.5) |
|  | 28-day mortality, n (%) | 30 (69.8) | 1080 (71.6) |  | 18 (64.3) | 1106 (71.9) |
|  | Length of hospital stay (days), median (IQR) | 13 (7–31) | 14 (8–23) |  | 13 (10–30) | 13 (8–23) |
| Survivor, (n) | | 5 | 269 |  | 5 | 272 |
| Survival rate (%) | | 11.6 | 17.8 |  | 17.9 | 17.7 |
|  | Length of hospital stay (days), median (IQR) | 31 (30–58) | 25 (15–46) |  | 30 (30–30) | 25 (15–46) |

PMX, polymyxin B-immobilised fibre column; mPSL, methylprednisolone; IPTW, inverse probability of treatment weighting; IQR, interquartile range
