# Supplementary material for: Polymyxin B-immobilised fibre column treatment for acute exacerbation of idiopathic pulmonary fibrosis patients with mechanical ventilation: a nationwide observational study
Source: J Intensive Care. 2023 Oct 11;11:45. doi: 10.1186/s40560-023-00693-0 (PMC10568810; doi:10.1186/s40560-023-00693-0)
Supplement: Supplementary file 6 — Additional file 6: Table S5. Comparison of outcomes between the PMX_S1 and mPSL alone_S1 groups after the stabilised IPTW in the sensitivity analyses 1. [file 40560_2023_693_MOESM6_ESM.docx]

**Additional file 6**

**Table S5.** Comparison of outcomes between the PMX_S1 and mPSL alone_S1 groups after the stabilised IPTW in the sensitivity analyses 1

| Logistic regression analyses of patients in the PMX_S1 and mPSL alone_S1 groups after the stabilised IPTW ^a^ | | | | |
| --- | --- | --- | --- | --- |
|  |  | Odds ratio | 95% CI | *p* value |
| All patients | |  |  |  |
|  | In-hospital mortality | 0.94 | 0.15–5.86 | 0.95 |
|  | 14-day mortality | 1.04 | 0.36–2.96 | 0.94 |
|  | 28-day mortality | 0.69 | 0.21–2.28 | 0.55 |
| Incidence rate ratios of length of hospital stay in the PMX_S1 and mPSL alone_S1 groups after the stabilised IPTW ^b^ | | | | |
|  |  | Incidence rate ratio | 95% CI | *p* value |
| All patients | |  | |  |
|  | Length of hospital stay | 1.18 | 0.74–1.88 | 0.49 |
| Survivors | |  | |  |
|  | Length of hospital stay | 0.94 | 0.79–1.11 | 0.47 |

PMX, polymyxin B-immobilised fibre column; mPSL, methylprednisolone; IPTW, inverse probability of treatment weighting; CI, confidence interval

^a^ The odds ratio of the PMX_S1 group compared to the mPSL alone_S1 group

^b^ The incidence rate ratio of the PMX_S1 group compared to the mPSL alone_S1 group
