# Supplementary material for: Polymyxin B-immobilised fibre column treatment for acute exacerbation of idiopathic pulmonary fibrosis patients with mechanical ventilation: a nationwide observational study
Source: J Intensive Care. 2023 Oct 11;11:45. doi: 10.1186/s40560-023-00693-0 (PMC10568810; doi:10.1186/s40560-023-00693-0)
Supplement: Supplementary file 7 — Additional file 7: Table S6. Baseline characteristics of the patients before and after the stabilised IPTW using propensity scores in the sensitivity analyses 2. [file 40560_2023_693_MOESM7_ESM.docx]

**Additional file 7**

**Table S6.** Baseline characteristics of the patients before and after the stabilised IPTW using propensity scores in the sensitivity analyses 2

|  | All patients | | |  | Patients after IPTW estimation | | |
| --- | --- | --- | --- | --- | --- | --- | --- |
| Characteristics | PMX_S2 group (n =195) | mPSL alone_S2 group (n = 5356) | SMD |  | PMX_S2 group (n = 192) | mPSL alone_S2 group (n =5372) | SMD |
| Male sex | 77% | 74% | 6.6 |  | 79% | 74% | 11.4 |
| Age, years |  |  |  |  |  |  |  |
| 51–70 | 38.5% | 25.0% | 29.3 |  | 25.4% | 25.4% | −0.1 |
| 71–80 | 43.6% | 45.0% | −2.9 |  | 41.3% | 45.1% | −7.7 |
| ≥80 | 17.9% | 30.0% | −28.5 |  | 33.4% | 29.5% | 8.3 |
| Treatment year |  |  |  |  |  |  |  |
| 2010–2012 | 35.4% | 29.5% | 12.6 |  | 30.4% | 29.6% | 1.6 |
| 2013–2015 | 38.5% | 35.5% | 6.2 |  | 35.5% | 35.6% | −0.1 |
| 2016–2018 | 26.2% | 35.0% | −19.3 |  | 34.1% | 34.8% | −1.5 |
| BMI (kg/m^2^) |  |  |  |  |  |  |  |
| <23 | 34.4% | 50.0% | −32.0 |  | 48.3% | 49.5% | −2.4 |
| ≥23 | 49.2% | 38.2% | 22.3 |  | 42.7% | 38.6% | 8.4 |
| Missing data | 16.4% | 11.8% | 13.3 |  | 9.0% | 11.9% | −9.6 |
| Hugh–Jones dyspnoea score upon admission | | | |  |  |  |  |
| 1–4 | 21.0% | 24.4% | −8.1 |  | 31.2% | 24.3% | 15.6 |
| 5 | 29.2% | 46.7% | −36.5 |  | 41.2% | 45.9% | −9.4 |
| Missing data | 49.7% | 28.9% | 43.6 |  | 27.5% | 29.8% | −5.0 |
| Japan Coma Scale score upon admission | | | |  |  |  |  |
| 0- or 1-digit (alert or dull) | 94.9% | 94.3% | 2.7 |  | 89.3% | 94.3% | −18.1 |
| 2-digit (somnolence) | 3.6% | 2.8% | 4.3 |  | 8.0% | 2.9% | 19.7 |
| 3-digit (coma) | 1.5% | 2.9% | −9.2 |  | 2.7% | 2.9% | −0.9 |
| Charlson Comorbidity Index | | | |  |  |  |  |
| 0 | 57.4% | 50.9% | 13.2 |  | 52.4% | 51.3% | 2.3 |
| 1 | 16.9% | 12.2% | 13.6 |  | 9.4% | 12.3% | −9.3 |
| 2 | 18.5% | 24.0% | −13.7 |  | 27.3% | 23.8% | 8.0 |
| ≥3 | 7.2% | 12.9% | −19.1 |  | 10.9% | 12.7% | −5.4 |
| Smoking index, pack-years | | | |  |  |  |  |
| 0 | 37.9% | 44.0% | −12.3 |  | 47.3% | 43.8% | 7.0 |
| 1–39 | 19.5% | 19.5% | −0.2 |  | 13.4% | 19.5% | −16.6 |
| ≥40 | 25.6% | 23.4% | 5.1 |  | 26.2% | 23.5% | 6.2 |
| Missing data | 16.9% | 13.0% | 10.9 |  | 13.2% | 13.2% | −0.1 |
| ADL upon admission (Barthel Index) | | | |  |  |  |  |
| 100 | 23.6% | 19.3% | 10.5 |  | 22.2% | 19.5% | 6.8 |
| ≤95 | 56.4% | 61.6% | −10.5 |  | 61.1% | 61.4% | −0.7 |
| Missing data | 20.0% | 19.1% | 2.2 |  | 16.7% | 19.1% | −6.3 |
| History of previous hospitalization | | | |  |  |  |  |
| 0 | 58.5% | 58.0% | 1.0 |  | 56.4% | 58.1% | −3.5 |
| 1–2 | 29.2% | 30.4% | −2.5 |  | 37.1% | 30.3% | 14.5 |
| ≥3 | 12.3% | 11.6% | 2.1 |  | 6.5% | 11.6% | −17.9 |
| Academic hospital | 82% | 83% | −1.6 |  | 87% | 83% | 10.1 |
| ICU admission | 46% | 26% | 41.7 |  | 21% | 27% | −12.9 |

Data are presented as n (%)

IPTW, inverse probability of treatment weighting; PMX, polymyxin B-immobilised fibre column; mPSL, methylprednisolone; SMD, standardised mean difference; BMI, body mass index; ADL, activities of daily living; ICU, intensive care unit
