# Supplementary material for: Polymyxin B-immobilised fibre column treatment for acute exacerbation of idiopathic pulmonary fibrosis patients with mechanical ventilation: a nationwide observational study
Source: J Intensive Care. 2023 Oct 11;11:45. doi: 10.1186/s40560-023-00693-0 (PMC10568810; doi:10.1186/s40560-023-00693-0)
Supplement: Supplementary file 8 — Additional file 8: Table S7. Comorbidities and treatments before and after the stabilised IPTW using propensity scores in the sensitivity analyses 2. [file 40560_2023_693_MOESM8_ESM.docx]

**Additional file 8**

**Table S7.** Comorbidities and treatments before and after the stabilised IPTW using propensity scores in the sensitivity analyses 2

|  | All patients | | |  | Patients after IPTW estimation | | |
| --- | --- | --- | --- | --- | --- | --- | --- |
| Variables | PMX_S2 group (n =195) | mPSL alone_S2 group (n = 5356) | SMD |  | PMX_S2 group (n = 192) | mPSL alone_S2 group (n =5372) | SMD |
| Comorbidity |  |  |  |  |  |  |  |
| Bronchial asthma | 5% | 5% | 1.9 |  | 3% | 5% | −8.2 |
| Chronic obstructive pulmonary disease | 3% | 5% | −11.5 |  | 2% | 5% | −15.2 |
| Pneumonia | 16% | 19% | −6.8 |  | 19% | 19% | 0.6 |
| Pulmonary embolism | 0% | 1% | −11.8 |  | 0% | 1% | −11.7 |
| Bronchiectasis | 2% | 3% | −4.6 |  | 3% | 3% | 0.0 |
| Pneumothorax | 1% | 1% | −3.0 |  | 1% | 1% | −0.4 |
| Lung cancer | 7% | 8% | −4.8 |  | 12% | 8% | 12.4 |
| Other types of cancer ^a^ | 4% | 8% | −17.2 |  | 5% | 7% | −9.1 |
| Disseminated intravascular coagulation | 20% | 9% | 32.7 |  | 14% | 9% | 14.4 |
| Chronic heart failure | 14% | 21% | −18.6 |  | 19% | 21% | −4.6 |
| Acute coronary syndrome | 5% | 8% | −12.2 |  | 8% | 7% | 2.0 |
| Diabetes mellitus | 25% | 27% | −4.9 |  | 29% | 27% | 4.6 |
| Stroke | 3% | 5% | −11.9 |  | 8% | 5% | 11.1 |
| Renal failure | 27% | 10% | 43.8 |  | 17% | 11% | 15.0 |
| Liver dysfunction | 5% | 5% | −2.1 |  | 6% | 5% | 2.6 |
| Gastroesophageal reflux disease | 8% | 13% | −16.2 |  | 12% | 12% | −0.7 |
| Urinary tract infection | 1% | 1% | 1.7 |  | 1% | 1% | 2.6 |
| Treatment within 3 days after hospitalisation | | | |  |  |  |  |
| Haemodialysis | 19% | 3% | 55.7 |  | 4% | 3% | 0.9 |
| High-flow nasal cannula oxygen therapy | 4% | 4% | −1.1 |  | 2% | 4% | −7.0 |
| Ampicillin/sulbactam | 9% | 14% | −14.3 |  | 16% | 14% | 6.3 |
| Tazobactam/piperacillin | 14% | 18% | −12.1 |  | 21% | 18% | 8.0 |
| Broad spectrum β-lactam antibiotics ^b^ | 67% | 52% | 30.4 |  | 53% | 53% | 0.2 |
| Fluoroquinolone | 49% | 32% | 36.1 |  | 26% | 33% | −13.2 |
| Anti-MRSA drug | 2% | 2% | 1.6 |  | 1% | 2% | −2.9 |
| Noradrenaline | 0% | 1% | −16.7 |  | 0% | 2% | −18.3 |
| Hydrocortisone | 2% | 2% | −4.8 |  | 1% | 2% | −8.5 |
| Cyclophosphamide (intravenous) | 9% | 3% | 26.9 |  | 3% | 3% | −0.3 |
| Tacrolimus | 2% | 1% | 5.3 |  | 1% | 1% | −3.4 |
| Pirfenidone | 4% | 2% | 11.8 |  | 1% | 2% | −7.3 |
| Nintedanib | 1% | 1% | −3.2 |  | 0% | 1% | −7.9 |
| Furosemide | 25% | 32% | −15.0 |  | 34% | 32% | 5.1 |

Data were presented as n (%)

IPTW, inverse probability of treatment weighting; PMX, polymyxin B-immobilised fibre column; mPSL, methylprednisolone; SMD, standardised mean difference; MRSA, methicillin-resistant *Staphylococcus aureus*

^a^ Detailed information in Additional file 2: Table S1

^b^ Third-generation cephalosporin, fourth-generation cephalosporin and carbapenem
