# Supplementary material for: Polymyxin B-immobilised fibre column treatment for acute exacerbation of idiopathic pulmonary fibrosis patients with mechanical ventilation: a nationwide observational study
Source: J Intensive Care. 2023 Oct 11;11:45. doi: 10.1186/s40560-023-00693-0 (PMC10568810; doi:10.1186/s40560-023-00693-0)
Supplement: Supplementary file 9 — Additional file 9: Table S8. Outcomes in the PMX_S2 and mPSL alone_S2 groups before and after the stabilised IPTW in the sensitivity analyses 2. [file 40560_2023_693_MOESM9_ESM.docx]

**Additional file 9**

**Table S8.** Outcomes in the PMX_S2 and mPSL alone_S2 groups before and after the stabilised IPTW in the sensitivity analyses 2

|  |  | Before the stabilised IPTW | |  | After the stabilised IPTW | |
| --- | --- | --- | --- | --- | --- | --- |
|  |  | PMX_S2 group | mPSL alone_S2 group |  | PMX_S2 group | mPSL alone_S2 group |
| All patients, (n) | | 195 | 5356 |  | 192 | 5372 |
|  | In-hospital mortality, n (%) | 156 (80.0) | 4105 (76.6) |  | 161 (83.9) | 4117 (76.6) |
|  | 14-day mortality, n (%) | 41 (21.0) | 1143 (21.3) |  | 47 (24.5) | 1145 (21.3) |
|  | 28-day mortality, n (%) | 95 (48.7) | 2626 (49.0) |  | 109 (56.8) | 2641 (49.2) |
|  | Length of hospital stay (days), median (IQR) | 28 (16–55) | 25 (15–45) |  | 23 (15–38) | 25 (15–44) |
| Survivor, (n) | | 39 | 1251 |  | 31 | 1255 |
| Survival rate (%) | | 20.0 | 23.4 |  | 16.1 | 23.4 |
|  | Length of hospital stay (days), median (IQR) | 58 (31–84) | 40 (25–62) |  | 52 (31–73) | 40 (25–62) |

PMX, polymyxin B-immobilised fibre column; mPSL, methylprednisolone; IPTW, inverse probability of treatment weighting; IQR, interquartile range
