# Supplementary material for: Polymyxin B-immobilised fibre column treatment for acute exacerbation of idiopathic pulmonary fibrosis patients with mechanical ventilation: a nationwide observational study
Source: J Intensive Care. 2023 Oct 11;11:45. doi: 10.1186/s40560-023-00693-0 (PMC10568810; doi:10.1186/s40560-023-00693-0)
Supplement: Supplementary file 10 — Additional file 10: Table S9. Comparison of outcomes between the PMX_S2 and mPSL alone_S2 groups after the stabilised IPTW in the sensitivity analyses 2. [file 40560_2023_693_MOESM10_ESM.docx]

**Additional file 10**

**Table S9.** Comparison of outcomes between the PMX_S2 and mPSL alone_S2 groups after the stabilised IPTW in the sensitivity analyses 2

| Logistic regression analyses of patients in the PMX_S2 and mPSL alone_S2 groups after the stabilised IPTW ^a^ | | | | |
| --- | --- | --- | --- | --- |
|  |  | Odds ratio | 95% CI | *p* value |
| All patients | |  |  |  |
|  | In-hospital mortality | 1.59 | 0.82–3.11 | 0.17 |
|  | 14-day mortality | 1.19 | 0.60–2.36 | 0.63 |
|  | 28-day mortality | 1.36 | 0.85–2.19 | 0.20 |
| Incidence rate ratios of length of hospital stay in the PMX_S2 and mPSL alone_S2 groups after the stabilised IPTW ^b^ | | | | |
|  |  | Incidence rate ratio | 95% CI | *p* value |
| All patients | |  | |  |
|  | Length of hospital stay | 0.94 | 0.78–1.14 | 0.54 |
| Survivors | |  | |  |
|  | Length of hospital stay | 1.18 | 0.82–1.69 | 0.39 |

PMX, polymyxin B-immobilised fibre column; mPSL, methylprednisolone; IPTW, inverse probability of treatment weighting; CI, confidence interval

^a^ The odds ratio of the PMX_S2 group compared to the mPSL alone_S2 group

^b^ The incidence rate ratio of the PMX_S2 group compared to the mPSL alone_S2 group
